# ViralBottleneck: an R package for estimating viral transmission bottlenecks from deep sequencing data using multiple methods

**DOI:** 10.1093/ve/veaf071

**Published:** 2025-09-19

**Authors:** Bowen Zheng, Paul C D Johnson, Joseph Hughes

**Affiliations:** MRC-University of Glasgow Centre for Virus Research, Sir Michael Stoker Building, 464 Bearsden Road, Glasgow G61 1QH, United Kingdom; School of Biodiversity, One Health and Veterinary Medicine, University of Glasgow, Graham Kerr Building, Glasgow G12 8QQ, United Kingdom; MRC-University of Glasgow Centre for Virus Research, Sir Michael Stoker Building, 464 Bearsden Road, Glasgow G61 1QH, United Kingdom

**Keywords:** bottleneck, intra-host viral diversity, R package, transmission, deep sequencing

## Abstract

Acute viral infections pose significant public health challenges. Since viral evolution, immune escape, and infection severity are influenced by how viruses spread between hosts, understanding transmission bottlenecks is crucial for predicting disease dynamics and developing effective control strategies. Transmission bottlenecks reduce viral population size and genetic diversity as the virus spreads to new hosts. Bottleneck size, defined as the number of viral individuals successfully establishing infection in a new host, varies across transmission events and can influence disease emergence and virus evolution. In this study, we introduce ViralBottleneck, an R package integrating six established methods for estimating transmission bottleneck size: the presence–absence method, Kullback–Leibler (KL) method, binomial method, two versions of the beta-binomial method, and the Wright–Fisher method. We demonstrate the package’s functionality using simulated datasets generated with SANTA-Sim under different scenarios with known bottleneck sizes. Our results reveal considerable variation in estimates across methods, highlighting the impact of methodological choice on bottleneck size estimation. The code and associated tutorial are available at https://github.com/BowenArchaman/ViralBottleneck.

## Introduction

Many serious health problems are caused by acute viral infections, for example, influenza infections and the novel severe acute respiratory syndrome coronavirus 2 (SARS-CoV-2) infections. Based on the World Health Organization (WHO) estimations, about 250 000 to 500 000 people die from influenza globally every year (Paget et al. 2019). In recent years, the outbreak of COVID-19, caused by SARS-CoV-2, has been a new global health problem. According to the statistics from the WHO, as of 2nd of February 2025, more than 777.4 million people have been affected and over 7 million people have died with COVID-19 (Word Health Organization 2023).

Understanding viral transmission dynamics is essential for infectious disease prevention and control. A key parameter in this process is the transmission bottleneck size, defined as the number of viral particles from the infecting individual (‘donor’) that successfully establish infection in the newly infected individual (‘recipient’) following transmission. Viral transmission bottlenecks—reductions in population size during host-to-host transmission—limit the diversity of variants entering the new host and thereby influence which lineages persist and propagate. This can have downstream effects on the rate of viral adaptation and on the types of mutations that become fixed or lost. During transmission, viruses face numerous barriers—such as mucosal defenses, immune factors, and physical barriers—which reduce the number of transmitted particles. As a result, the smaller viral population in the recipient shapes the genetic composition of subsequent generations, potentially limiting adaptive potential. For example, transmission bottlenecks may slow the fixation of beneficial mutations or disrupt the co-transmission of epistatically interacting variants. However, bottlenecks can also benefit viral populations by purging defective or ‘cheater’ genomes (e.g. defective interfering particles), thereby restoring population fitness over time (Zwart and Elena 2015). This dual role makes bottlenecks an important evolutionary filter that can either constrain or facilitate long-term viral success depending on context. Experimental and theoretical studies have demonstrated the evolutionary significance of bottlenecks. In vesicular stomatitis virus, smaller inoculum sizes were associated with increased genetic drift and altered virulence trajectories (Novella et al. 1995). Similarly, theoretical work has suggested that reduced population sizes at transmission may limit virulence and adaptability (Ebert 1998, Yuste et al. 1999). In the case of SARS-CoV-2, tight transmission bottlenecks have been observed. This suggests that within-host selection, rather than inter-host transmission dynamics, may be the primary force driving viral evolution for SARS-CoV-2 (Bendall et al. 2023). These findings also imply that chronic infections—where selection can act over longer timescales—may play a disproportionate role in the emergence of new variants (Lythgoe et al. 2021). Quantifying transmission bottlenecks can enable comparisons of how different transmission routes impact viral evolution (Frise et al. 2016), and inform assessments of infection risk and cross-species emergence (Geoghegan et al. 2016). Thus, quantifying transmission bottlenecks is essential not only for understanding viral evolution but also for improving epidemiological models, anticipating the dynamics of variant emergence, and informing surveillance strategies.

Owing to the strong influence of population bottlenecks on viral genetic diversity, much attention has been paid to modeling this process to estimate the size of the bottleneck (i.e., the number of viral genomes from the donor that establishes in the recipient), resulting in the development of several bottleneck estimation methods that use data from deep sequencing. Early studies used clonal Sanger sequencing of amplicons from the viral population (Bull et al. 2011), but the advent of high-throughput sequencing has enabled the investigation of much finer-scale viral population diversity. Despite constant improvements in sequencing technologies, there are still biases introduced during the process of sequencing, such as systematic errors and sequencing errors (polymerase bias and incorporation of sequencing errors) (Robasky et al. 2014). Therefore, to distinguish the true variants from the sequencing errors, it is common to develop analytical pipelines to call reliable variants and to introduce a suitable variant calling threshold to filter out sequencing errors.

Several statistical approaches have been developed for bottleneck estimation: the adaptation of the Wright–Fisher model (Wright 1931) for influenza virus (Poon et al. 2016); Kullback–Leibler divergence (Kullback and Leibler 1951) for ebolavirus (Emmett et al. 2015); the presence–absence model for tobacco mosaic virus (Sacristán et al. 2003); and more recently, binomial and beta-binomial models for influenza virus (Sobel Leonard et al. 2017b). However, these methods have not yet been integrated into a single tool for measuring transmission of bottleneck sizes. Such a tool could facilitate exploring factors influencing transmission bottleneck quantification. In addition, this tool could aid comparison between methods on the same data set and shed light on the comparison of bottleneck sizes between studies.

Here, we present an R package implementing six methods that can be applied to measuring transmission bottleneck sizes, and we compare the estimates of each method on simulated data sets. For creating the simulated datasets, two important factors that we expect to influence bottleneck size estimation were varied: the average coverage of the deep-sequencing data (i.e. sequencing depth) and the number of generations since the founder viral population in the recipient. To demonstrate the potential of the R package and evaluate the current methods under these conditions, we simulated transmission bottlenecks of varying sizes across different levels of sequencing depth and time intervals since the transmission bottleneck event.

### Product

To facilitate the estimation of bottleneck using intra-host single nucleotide variants (iSNVs), we developed an R package called ‘ViralBottleneck’ (https://github.com/BowenArchaman/ViralBottleneck). The package implements six established methods: Wright–Fisher, Kullback–Leibler (KL) divergence, presence–absence, binomial, beta-binomial approximate, and beta-binomial exact. A user tutorial is provided in the github repository. Table 1 summarizes the assumptions underlying each method and the evolutionary processes they model, to help users identify the approach most suitable for their dataset. Mathematical details of the implemented methods are provided in Supplementary File S1.

**Table 1:** Summary table of the assumptions that the methods make.

Method	Allows single pairs	Usage of variant frequency in recipient	Models post-bottleneck growth	Models sequencing depth	Models sequencing error	Allows multi-allelic sites	Robust to non-neutral sites
Presence–absence	✓	✗	✗	✗	✗	✗	✓
KL	✓	✓	✗	✗	✗	✓	✗
Binomial	✓	✓	✗	✓	✓	✗	✗
Beta-binomial approximate	✓	✓	✓	✗	✓	✗	✗
Beta-binomial exact	✓	✓	✓	✓	✓	✗	✗
Wright–Fisher	✗	✓	✗	✗	✗	✓	✗

**Figure 1:**
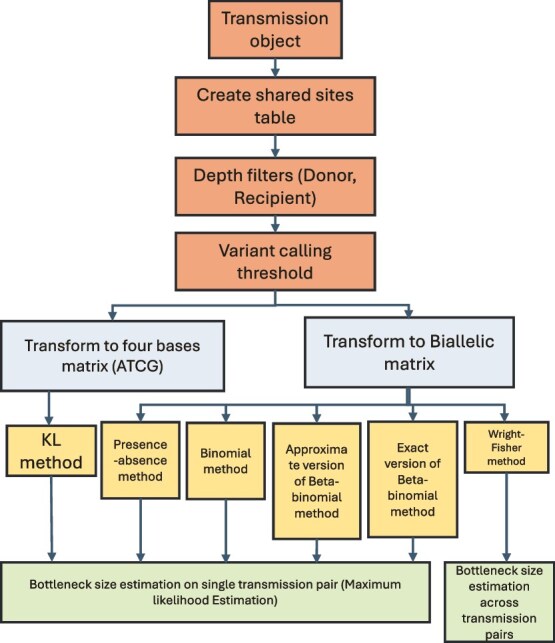
Flow chart describing the main processes in the package.

The main components in the R package are shown in Fig. 1. ‘CreateTransmissionObject’ takes transmission pair names as input and loads the deep-sequencing data for each pair from the working directory. The input file is in the comma-separated value format (‘.csv’) and is illustrated in Table 2. The package allows for the user to choose whether all, only synonymous, or non-synonymous variant sites are used in the calculations. This enables the user to explore the effect of selection on the estimates of bottleneck sizes, especially as the methods implemented in the package all assume neutral selection.

**Table 2:** Example of sample csv file as input for the transmission object creation.

Pos	Segment	A	T	C	G	Non/Syn
207	1	12 544	0	0	2 504	Syn
228	1	0	505	13 445	0	Syn
303	1	4 539	0	0	172	Syn
360	1	0	128	4 059	0	Syn
700	1	0	383	2 680	0	Syn
1 128	1	7 027	0	0	318	Syn
1 527	1	12 728	0	0	1 032	Syn
1 644	1	0	1 538	5 183	0	Syn

*Note.* The first column, *Pos*, gives the position of each variant site on the viral segment or genome. The second column gives the *segment* number. The frequency of each base in the deep-sequencing data is given in columns three to six. The last column (*Non/Syn*) records whether the variant is synonymous or non-synonymous.

**Figure 2:**
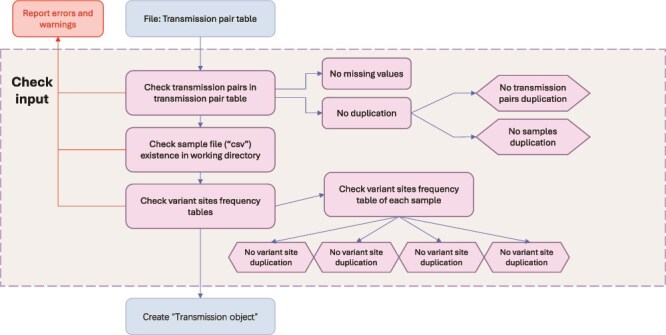
Flow chart illustrating the input function checks.

**Figure 3:**
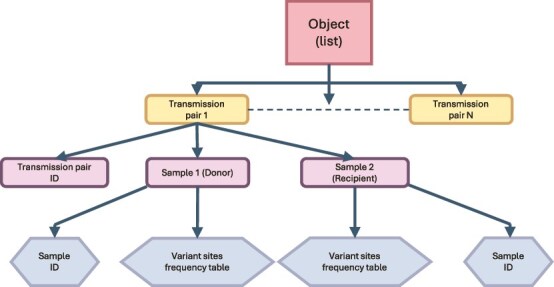
The structure of the transmission object.

Before creating the transmission object, the inputs are checked. As Fig. 2 shows, the transmission pair table, which contains the names of donors in the first column and recipients in the second column, is input to the ‘check’ function. In the ‘check input’ block, the first step is to check the transmission pair table itself to avoid missing values and duplicated transmission pairs in the table which could influence subsequent steps. The package will only issue a warning if donor or recipient samples are duplicated, as long as the pair is not duplicated, in which case it raises an error and exits. Then, based on the sample names in the transmission pairs table, the program will take the sample file (which is in csv format) to check that the table dimensions meet the requirements, check that there are no missing values, that each column content meets the requirements and check whether variant sites are duplicated. Finally, only when all the conditions meet the requirements will a transmission object be created with the data. As Fig. 3 shows, the transmission object is a list of transmission pairs. It can be used as a ‘list’ in R to extract pairs using indexing. ‘Transmission pair’ is an R object class. It contains the transmission pair ID that is created by linking the donor and recipient sample names with a ‘-’ character, and two ‘sample’ R object classes: donor and recipient. The ‘sample’ data structure stores the sample ID and the variant sites table containing the following information in columns (Table 2): position along the genome, viral genome segment name, frequencies of the four bases (A, C, G, and T), and whether the allele of the variant site are synonymous or non-synonymous mutations. The input for calculating bottleneck size is called a transmission object.

Four of the five bottleneck estimation methods (all except the KL method) need the frequencies of the four bases merged into two categories (biallelic). Therefore, a unified biallelic matrix is created containing frequencies of the minority and majority variant in the donors and the recipients. To generate the biallelic matrix, the frequencies of the four bases are sorted and the base with the greatest frequency in the donor is selected as the dominant base for the site. The base with the second greatest frequency is checked to determine whether the second frequency is equal to the third frequency to make sure that the site is biallelic. If they are equal, the site is removed. Then, the second frequency in the donor is checked to determine whether it is greater than the variant calling threshold. The numbers of variants used and filtered in the calculation are reported in the log file. As Fig. 2 shows, the bottleneck size calculation takes the ‘transmission object’ as input. A table containing the shared iSNVs between donors and recipients of a transmission pair is created. The variant sites in the donor or the recipient with lower depth coverage than the depth threshold set by the user are filtered out. Subsequent steps depend on the method used. For all methods except the KL method, the dominant and subdominant bases are selected and then the variant calling threshold for the subdominant variant in the donor is applied. Following filtering, bottleneck size is estimated based on the final matrix. The calculation of the beta-binomial method was validated against the previously published code (see Supplementary Tables S1 and S2). For the KL method, there are no steps for selecting dominant and subdominant bases. The KL method can use the frequencies of the four bases at variable sites to quantify the bottleneck size. The Wright–Fisher method differs substantially from the other methods as it requires multiple transmission pairs as input. Before using this method, the transmission object must contain more than one transmission pair.

### Methodology for the simulations

**Figure 4:**
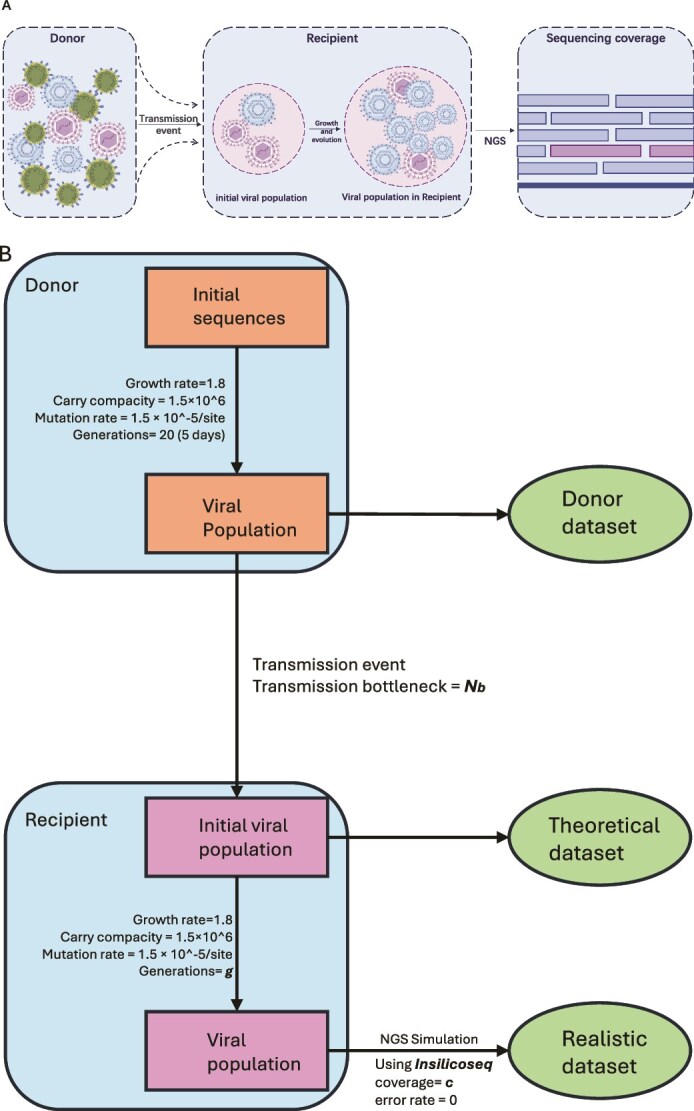
The flow chart of simulation. (A) Overview of the simulation approach. (B) Flow chart of the simulations based on the general models shown in Fig. 1A. The relevant parameters are listed next to the arrows.

In order to illustrate the use of the R package, we simulated a limited set of scenarios with simplifying assumptions, including the absence of selection, reassortment, and sequencing errors. The approach used for data simulation and the analytical procedure are shown in Fig. 4. The data simulation contains four parts: evolution of the viral population in the donor from one sequence, the bottleneck event, growth of viral population in the recipient, and two scenarios of obtaining datasets from recipients.

To create a viral population in the donor from one single sequence, SANTA-Sim (Jariani et al. 2019) was used to simulate the growth and evolution of viral genomes. The eight segments of the influenza A virus, H1N1/California (A/California/07/2009(H1N1)) were concatenated to create the initial genome sequence, thereby assuming no within-host reassortment. While this represents a simplification, previous studies have reported limited reassortment within host (Sobel Leonard et al. 2017a), supporting the simplifying assumption for the purposes of simulation. The donor population was simulated using SANTA-sim, assuming a mutation rate 1.5e-05/site (Parvin et al. 1986), a reproductive rate of 1.8/generation (Hadjichrysanthou et al. 2016), and a carrying capacity of 1.5e6 (Baccam et al. 2006). All mutations are assumed to be neutral. The initial viral sequences in the donor underwent growth and evolution for 20 generations and formed the donor viral population. For IAV, a within-host generation time of ~ 6 h is commonly used, meaning that 20 generations correspond to roughly 5 days post-infection (Baccam et al. 2006). The number of iSNV > 0.01 and 0.02 are 25 and 7, respectively, in the simulated viral population (see Supplementary Fig. S2), which is consistent with previous IAV studies (McCrone et al. 2018). In the calculation, we set 0.01 as the variant calling threshold. That is, we assume the variants below this threshold could not be detected in donors and recipients. The ‘sampler’ module from SANTA-Sim provided whole population sequences as FASTA files. We converted the variation observed in the FASTA files to the input format for ViralBottleneck.

For the bottleneck size simulation, we used the ‘sampler’ model from SANTA-Sim to sample founder populations of size 1, 10, 20, 30, 40, and 50 sequences from the simulated donor viral population. These sequences formed the founder viral populations in the recipients. Their sample sizes represent the ‘true’ bottleneck size, which we refer to as the theoretical dataset, as in practice it would be impossible to sample these sequences. Each simulation of the same bottleneck size was repeated five times to assess bottleneck estimation consistency.

After the simulation of the bottleneck event, we simulate viral population growth and evolution in the recipient using the same parameters as in the donor. Finally, we simulated two scenarios to obtain datasets of viral genomes from recipients. In the first scenario, we directly obtained information of the initial viral particles without growth and evolution in the recipient; that is, we directly transform the FASTA format sequence file obtained from SANTA-Sim into the input format for the package ViralBottleneck. In the second scenario, the initial viral particles in the recipient underwent growth and evolution for several generations using the same parameters as in the donor (see growth curves in Supplementary Fig. S1). The genome sequences of the whole viral population in the recipient at different generations post-bottleneck were obtained as FASTA files. InSilicoSeq (Gourlé et al. 2019) was used to simulate the deep-sequencing process using these FASTA files as input and applied the perfect error mode (no sequencing errors introduced). We mapped reads using bowtie2 and called all the variants using DiversiTools without filtering (https://github.com/josephhughes/DiversiTools). The output of DiversiTools was transformed into the input format for the R package.

As a result, four simulated datasets were created by varying the simulation parameters as described in Table 3. As shown in the flow chart, the datasets were divided into two categories. The dataset created directly from the compilation of mutation frequencies from the initial viral population was named ‘Theoretical simulated dataset’, because it is unlikely that this viral population could ever be sampled *in vivo*. The dataset created from the simulation following viral population growth and evolution was named ‘Realistic simulated dataset’, because it reflects the growth and deep-sequencing processes, which are more ‘realistic’.

**Table 3:** Parameters of simulated datasets.

Sampling type	Dataset name	Transmission bottleneck	Post-bottleneck	Average coverage
Theoretical	‘Bottleneck-T’	1–50	0 days	1–50
Realistic	‘Generation’	50	1–5 days	10 000
Realistic	‘Coverage’	50	3 days	10–10 000
Realistic	‘Bottleneck-R’	1–50	3 days	2 000

*Note.* The table shows the parameters varied between data sets: the size of the bottleneck, the number of generations of growth, and the sequencing coverage.

## Results

**Figure 5:**
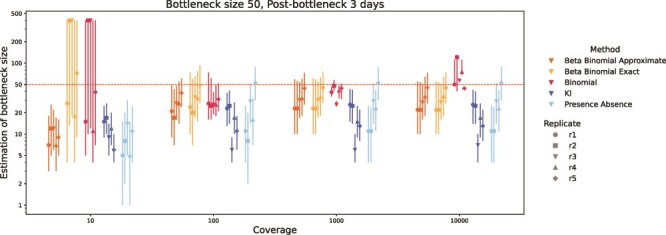
The influence of coverage on transmission bottleneck size estimation of different methods. The x-axis is the average coverage in the deep-sequencing simulated process. The y-axis is the logarithm to base e of estimated bottleneck size. Colors represent methods, and the point shapes represent sample replicates. Vertical lines extending from the points represent 95% confidence intervals. The horizontal dotted line is the true bottleneck size.

For most methods, varying the coverage from 10 to 10 000 had little impact on bottleneck size estimates (Fig. 5). However, the precision of estimates, as reflected in the width of the 95% confidence intervals (CIs), improved with increasing coverage—particularly for the binomial and exact beta-binomial methods. The approximate beta-binomial showed increased accuracy and reduced variability with higher coverage, while the exact version remained consistently accurate across all coverage levels. In terms of fluctuation in the results of the calculations between replicates, the results from the exact and approximate beta-binomial method and the binomial method become more precise and consistent with greater depth. For the presence–absence method and KL method, the estimate increased when the average coverage changed from 10 to 100, but had lower accuracy compared to the other methods. Overall, the estimates were stable and highly consistent across the coverage levels of 100, 1000, and 10 000.

**Figure 6:**
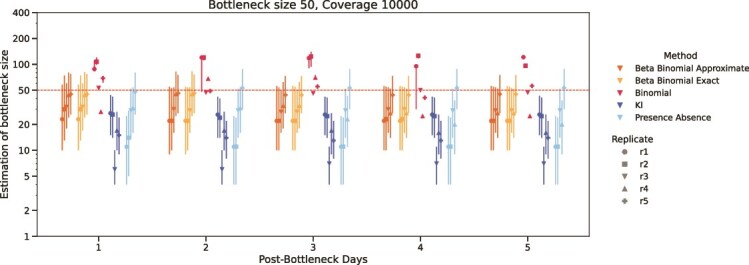
The influence of viral population generations on transmission bottleneck size estimation of different methods. The x-axis is the number of generations post-transmission. The y-axis is the logarithm to base e of estimated bottleneck size, with results shown for different post-bottleneck days. Colors represent methods, and the point shapes represent sample replicates. Vertical lines extending from the points represent 95% confidence intervals. The horizontal dotted line is the true bottleneck size.

Estimates of transmission bottleneck sizes based on the number of generations post-transmission are shown in Fig. 6. The larger the number of generations, the more the virus has undergone growth and evolution in the recipient. The estimated bottleneck sizes exhibited minimal variation across increasing numbers of post-transmission generations for all methods. The beta-binomial approach consistently outperformed other methods in terms of estimation accuracy. In addition, we find unexpectedly narrow and occasionally asymmetrical CIs occurred using the binomial method with coverage of ≥ 1000 (Fig. 5). These anomalous CIs were generated due to multimodal likelihood curves that occur because of the method’s assumption that the variant frequencies do not change between infection and sampling (Sobel Leonard et al. 2017b). Therefore, users should take the unreliability of the CIs generated by the binomial method into consideration when choosing a method.

**Figure 7:**
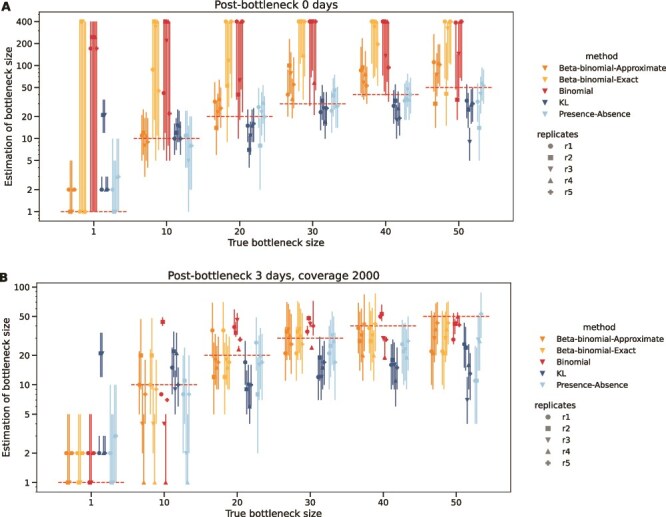
Comparison of bottleneck size estimates using the theoretical ‘bottleneck-T’ (A) and realistic ‘bottleneck-R’ (B) data sets. The x-axis shows the true simulated bottleneck sizes. The y-axis shows the logarithm to base e of estimated bottleneck size. Colors represent methods and point shapes represent replicates. The horizontal dotted lines show the true bottleneck size used in the test. Vertical lines extending from the points represent 95% confidence intervals. The vertical dotted lines are used to distinguish different bottleneck size groups. Bottleneck sizes were estimated in the range of 1–400, and in some cases the bottleneck was estimated at the maximum value or was not shown if a maximum-likelihood estimate was not found in the range.

Figure 7A illustrates the comparison of bottleneck size methods on the ‘Bottleneck-T’ dataset. The estimations of the KL method and the presence–absence method are more accurate than other methods and change along with the bottleneck sizes. However, the approximate version of beta-binomial method overestimates bottleneck size in the ‘Bottleneck-T’ dataset. In addition to having large CIs, the exact beta-binomial and the binomial frequently overestimate the bottleneck sizes. Their CIs only occasionally overlap with the true simulated values. Figure 7B shows the estimation comparison of different bottleneck sizes using different methods in the ‘Bottleneck-R’ dataset. For the smaller bottleneck sizes (e.g. 10 and 20), most methods produce estimates close to the true bottleneck size. However, for bigger bottleneck sizes (e.g. 50), the results show a trend toward underestimating the bottleneck size.

## Discussion and conclusion

We developed an R package ViralBottleneck, which implements six methods for estimating viral bottleneck sizes from deep-sequencing data. We demonstrated how to apply the package and compare different methods for bottleneck size estimation under various simulation scenarios with known bottleneck sizes. Our results show the estimates vary considerably using different methods on the same datasets. When using the ‘theoretical’ dataset, the presence–absence method and the KL method estimate the bottleneck sizes the most accurately. The remaining three methods (binomial, approximate beta-binomial, and exact beta-binomial) perform similarly, and better than the KL and presence–absence method on ‘realistic’ datasets. Our results also show that the timing of sampling after the transmission bottleneck does substantially affect the estimated bottleneck size. However, potential biases may still be present, as the models do not account for all post-bottleneck dynamics in the recipient. In particular, host immune responses can sharply reduce the viral population shortly after infection (Baccam et al. 2006), potentially introducing additional intra-host bottlenecks. These events could obscure the original transmission signal and influence bottleneck size estimation.

There are some limitations to this study. In the simulation, we concatenated the eight influenza viral segments into one sequence and ignored that some viruses have multi-segments on transmission. Mutation rates differ between the different viral segments, and this was not simulated. Finally, our simulation does not include sequencing errors. Nonetheless, this simplified simulation was sufficient to illustrate how the package can be used to test different scenarios and compare the performance of different approaches for bottleneck size estimation.

Currently, the ViralBottleneck package includes only methods that use iSNVs that are shared between donors and recipients. We assume that the shared iSNVs between recipients and donors are caused by transmission events. Previous studies found that shared variants in recipients may recur in hosts over time, due to parallel evolution (Braun et al. 2021), sequencing error (Worby et al. 2017), and homoplasy (Martin and Koelle 2021). However, the work conducted on SARS-CoV-2 (Braun et al. 2021) and IAV (McCrone et al. 2018) suggests minority variants were rarely shared among hosts. Therefore, the estimation accuracy is likely to only be affected by recurring variants when there are very few iSNVs used in the calculation.

In addition, not all the existing methods are compiled into this R package as some methods need different input formats. Future improvements will involve adding more methods, such as a method applying haplotype structure (Ghafari et al. 2020), a framework accounting for identification of variants under selection (Lumby et al. 2018) and a method using clonal variants (Shi et al. 2024). Our goal in the next phase of development is to create a unified framework that ensures easy comparison of all the methods and allows users to select the most suitable method based on their available data.

## Supplementary Material

supplementary_file1_veaf071

supplementary_file1(mathematical_model)_veaf071
