# Correction to: ViralBottleneck: an R package for estimating viral transmission bottlenecks from deep sequencing data using multiple methods

**DOI:** 10.1093/ve/veag055

**Published:** 2026-08-14

This is a correction to: Bowen Zheng, Paul C D Johnson, Joseph Hughes, ViralBottleneck: an R package for estimating viral transmission bottlenecks from deep sequencing data using multiple methods, *Virus Evolution*, Volume 11, Issue 1, 2025, veaf071, https://doi.org/10.1093/ve/veaf071

In the originally published version of this manuscript, the original Supplementary Material file contained an error in one of the formulae. In section 3, “2var” should be replaced with “var” due to the haploid nature of the data.

This error has been corrected and the Supplementary Material has been resupplied.

