# Supplementary material for: ViralBottleneck: an R package for estimating viral transmission bottlenecks from deep sequencing data using multiple methods
Source: Virus Evol. 2025 Sep 19;11(1):veaf071. doi: 10.1093/ve/veaf071 (PMC12516950; doi:10.1093/ve/veaf071)
Supplement: supplementary_file1_veaf071 [file supplementary_file1_veaf071.docx]

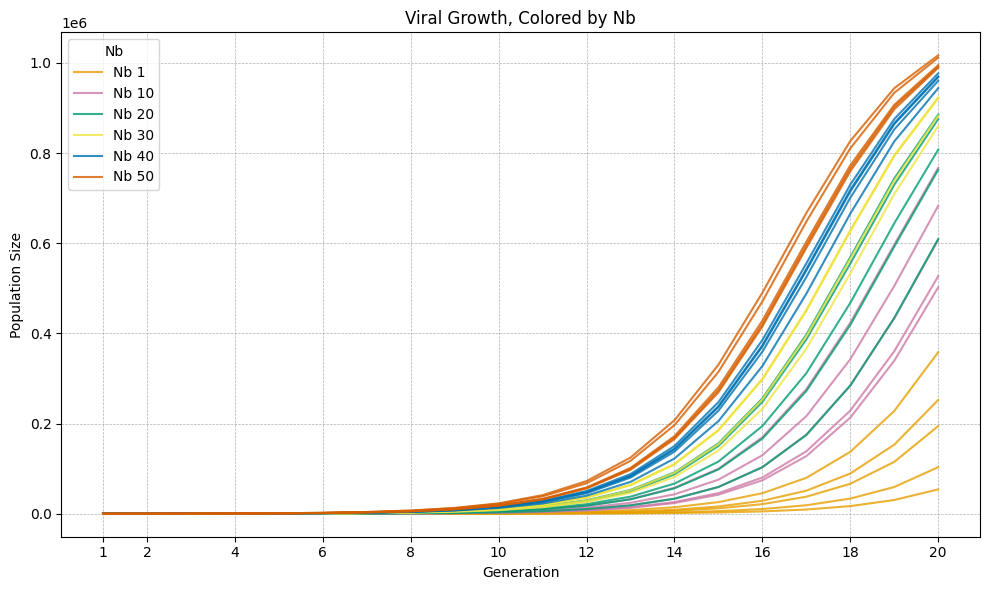


**Figure S1. Plot of the size of viral population at different generation.** The y-axis shows the population sizes. The x-axis shows different generations. Different colors represent viral population starting from different initial population sizes.

s


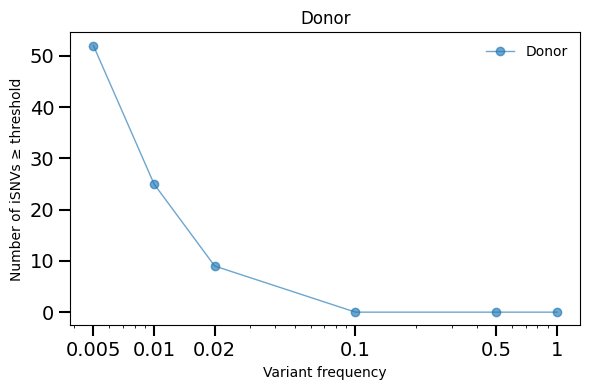


**Figure S2. Plot of the number of iSNV greater than different variant frequency thresholds from the viral population in the donor.** The y-axis shows the number of iSNV. The x-axis shows different variant frequency thresholds.

**Supplementary tables and figures**

**Table S1. Comparison between ViralBottleneck and BB_bottleneck transmission bottleneck estimates using the approximate beta-binomial bottleneck methods.**

| Donors | Recipients | ViralBottleneck estimate [95%CI] | BB_bottleneck estimate [95%CI] |
| --- | --- | --- | --- |
| Donor_3000 | 50_3_All_r1 | 45 [30-64] | 45 [30-64] |
| Donor_3000 | 50_6_All_r1 | 28 [20-39] | 28 [20-39] |
| Donor_3000 | 50_9_All_r1 | 34 [23-47] | 34 [23-47] |
| Donor_3000 | 50_12_All_r1 | 47 [31-67] | 47 [31-67] |
| Weissmanlab_donor | Weissmanlab_  recipient | 54 [48-61] | 54 [48-61] |

*Note.* The first four transmission pairs are from simulated datasets. The last transmission pairs are from example dataset in <https://github.com/weissmanlab/BB_bottleneck>.

**Table S2. Comparison between ViralBottleneck and BB_bottleneck transmission bottleneck estimates using the exact beta-binomial bottleneck methods.**

| Donors | Recipients | *ViralBottleneck* estimate [95%CI] | BB_bottleneck estimate [95%CI] |
| --- | --- | --- | --- |
| Donor_3000 | 50_3_All_r1 | 55 [34-90] | 55 [34-90] |
| Donor_3000 | 50_6_All_r1 | 33 [22-49] | 33 [22-49] |
| Donor_3000 | 50_9_All_r1 | 40 [26-61] | 40 [26-61] |
| Donor_3000 | 50_12_All_r1 | 56 [35-92] | 56 [35-92] |
| Weissmanlab_donor | Weissmanlab_recipient | 56 [49-63] | 56 [49-63] |

*Note.* The first four transmission pairs are from simulated datasets. The last transmission pair is from the example dataset in <https://github.com/weissmanlab/BB_bottleneck>.
