# Supplementary material for: ViralBottleneck: an R package for estimating viral transmission bottlenecks from deep sequencing data using multiple methods
Source: Virus Evol. 2025 Sep 19;11(1):veaf071. doi: 10.1093/ve/veaf071 (PMC12516950; doi:10.1093/ve/veaf071)
Supplement: supplementary_file1(mathematical_model)_veaf071 [file supplementary_file1(mathematical_model)_veaf071.docx]

**Mathematical models**

**1.Basic terms**

**Shared variants**: Variable sites observed in the donor and the recipient.

**Present, absent**: In the Presence-Absence method, Binomial method and both Beta-binomial methods, a variant in the donor that is detected in the recipient is described as present, and if it is not detected in the recipient it is described as absent (low frequency variants in the recipient can be generated by sequencing errors, but are generally filtered out as a result of the variant calling threshold).

| Terms | Description |
| --- | --- |
| $\boldsymbol{N}_{\boldsymbol{b}}$ | Transmission bottleneck size in estimation |
| $\boldsymbol{L}\left( \boldsymbol{N}_{\boldsymbol{b}} \right)$ | Likelihood vector for parameter Nb estimation |
| $\boldsymbol{i}$ | Index of variant sites |
| $\boldsymbol{D}$ | Donor in one transmission pair |
| $\boldsymbol{R}$ | Recipient in one transmission pair |
| $\boldsymbol{D}_{\boldsymbol{d}}$ | Dominant variant at site in the donor |
| $\boldsymbol{D}_{\boldsymbol{s}}$ | Sub-dominant variant at site in the donor |
| $\boldsymbol{R}_{\boldsymbol{d}}$ | Dominant variant at site in the recipient |
| $\boldsymbol{R}_{\boldsymbol{s}}$ | Sub-dominant variant at site in the recipient |
| $\boldsymbol{P}$ | Proportion of the base at a variant site |
| $\boldsymbol{f}$ | Frequency of the base at a variant site |
| $\boldsymbol{n}$ | The number of shared sites |
| $\boldsymbol{n}_{\boldsymbol{ab}}$ | The number of variants which are absent in the recipient |
| $\boldsymbol{n}_{\boldsymbol{pre}}$ | The number of variants which are present in the recipient |
| $\boldsymbol{i}_{\boldsymbol{ab}}$ | Index of absent variant sites used in calculation |
| $\boldsymbol{i}_{\boldsymbol{pre}}$ | Index of present variant sites used in calculation |

**Table 1. Introduction of basic terms for formula**

**2. Method for an individual donor-recipient pair**

2.1 Presence-Absence method

The Presence-Absence method (Sacristán et al., 2011; Sacristán et al., 2003) used the simplest model applied on deep sequencing data to estimate bottleneck size by modelling the probability that a viral variant will be transmitted from donor to recipient. This model uses variant frequencies in the donor and then uses information of the presence/absence of these variants in the recipient to estimate bottleneck size. The Presence-absence model is:

$$L(N_{b})=\prod_{i_{ab}=1}^{n_{ab}} {(1-\boldsymbol{P}_{\boldsymbol{D}_{\boldsymbol{s}}\boldsymbol{,}\boldsymbol{i}_{\boldsymbol{ab}}})}^{N_{b}}\prod_{i_{pre}=1}^{n_{pre}} [{1-(1-\boldsymbol{P}_{\boldsymbol{D}_{\boldsymbol{s}}\boldsymbol{,}\boldsymbol{i}_{\boldsymbol{pre}}})}^{N_{b}}]$$

$\boldsymbol{P}_{\boldsymbol{D}_{\boldsymbol{s}}\boldsymbol{,}\boldsymbol{i}_{\boldsymbol{ab}}}$ are the sub-dominant variant’s proportions on sites which are absent in the recipient. $\boldsymbol{P}_{\boldsymbol{D}_{\boldsymbol{s}}\boldsymbol{,}\boldsymbol{i}_{\boldsymbol{pre}}}$ are the sub-dominant variant’s proportions on sites which are present in the recipient. $N_{b}$ is the bottleneck size parameter. $L(N_{b})$ is the likelihood of $N_{b}$.

2.2. KL method:

Compared with the Presence-Absence method, KL method (Emmett et al., 2015) uses both variants’ frequencies in donor and variants’ frequencies in recipients to quantify the bottleneck size. The viral transmission in this method is modelled as binomial process. This method introduced KL divergence applying Stirling’s approximation on original binomial formula. The KL method model is:

$$L(N_{b})=-N_{b}\sum_{i=1}^{S} KL\left( \boldsymbol{P}_{\boldsymbol{D,i}} | \boldsymbol{P}_{\boldsymbol{R,i}} \right)+\frac{S}{2}log(N_{b})$$

$\boldsymbol{P}_{\boldsymbol{D,i}}$ is the set of four nucleotide frequencies in the donor on site $i$. $\boldsymbol{P}_{\boldsymbol{R,i}}$ is the set of nucleotide frequencies in the recipient at site $i$. $KL\left( \boldsymbol{P}_{\boldsymbol{D,i}} | \boldsymbol{P}_{\boldsymbol{R,i}} \right)$ is **Kullback–Leibler** divergence from $\boldsymbol{P}_{\boldsymbol{D,i}}$ to $\boldsymbol{P}_{\boldsymbol{R,i}}$ (Kullback & Leibler, 1951). *S* is the number of the shared variants between the donor and the recipient. $N_{b}$ is the bottleneck size in parameter estimation in MLE. $L(N_{b})$ is the log likelihood of the $N_{b}$.

2.3 Binomial method:

The binomial method (Emmett et al., 2015; Poon et al., 2016), compared to the previous method, not only uses information of proportions of shared variants in donors and recipients but also accounts for binomial sampling due to next generation sequencing, and models low frequency sequencing errors. The variants are divided into two groups: sites with variant proportions higher than the variant-calling threshold, and those with variant proportions lower than the variant-calling threshold, in order to reduce the effect of sequencing errors. The binomial model is:

$$n=n_{pre}+n_{ab}$$

$$log(L(N_{b}))=\sum_{i_{pre}=0}^{n_{pre}} {log(L(N_{b})}_{i_{pre}})+\sum_{i_{ab}=0}^{n_{ab}} {log(L(N_{b})}_{i_{ab}})$$

Present:

$${L(N_{b})}_{\boldsymbol{i}_{\boldsymbol{pre}}}=\sum_{k=0}^{N_{b}} P\_bin(\boldsymbol{f}_{\boldsymbol{R}_{\boldsymbol{s}}\boldsymbol{,}\boldsymbol{i}_{\boldsymbol{pre}}}|\boldsymbol{f}_{\boldsymbol{R}_{\boldsymbol{total}}\boldsymbol{,}\boldsymbol{i}_{\boldsymbol{pre}}}, \frac{k}{N_{b}})P\_bin(k|N_{b}, \boldsymbol{P}_{\boldsymbol{D}_{\boldsymbol{s}}\boldsymbol{,}\boldsymbol{i}_{\boldsymbol{pre}}})$$

Absent:

$${L(N_{b})}_{\boldsymbol{i}_{\boldsymbol{ab}}}=\sum_{k=0}^{N_{b}} P\_bin\_cdf(\boldsymbol{f}_{\boldsymbol{R}_{\boldsymbol{s}}\boldsymbol{,}\boldsymbol{i}_{\boldsymbol{ab}}}<T\boldsymbol{f}_{\boldsymbol{R}_{\boldsymbol{total}},\boldsymbol{i}_{\boldsymbol{ab}}}|\boldsymbol{f}_{\boldsymbol{R}_{\boldsymbol{total}},\boldsymbol{i}_{\boldsymbol{ab}}}, \frac{k}{N_{b}})P\_bin(k|N_{b}, \boldsymbol{P}_{\boldsymbol{D}_{\boldsymbol{s}}\boldsymbol{,}\boldsymbol{i}_{\boldsymbol{ab}}})$$

${L(N_{b})}_{\boldsymbol{i}_{\boldsymbol{pre}}}$ is the likelihood of bottleneck size $N_{b}$ for variants at sites $i_{pre}$, which are present in the recipient. $k$ is the number of viral particles passing through the bottleneck that carry the sub-dominant variant, and $N_{b}-k$ is the number that carry the dominant variant. The maximum value of $k$ is therefore $N_{b}.$ $\boldsymbol{f}_{\boldsymbol{R}_{\boldsymbol{s}}\boldsymbol{,}\boldsymbol{i}_{\boldsymbol{pre}}}$ is the number of reads at site $\boldsymbol{i}_{\boldsymbol{pre}}$ with the sub-dominant variant present in the recipient, while $\boldsymbol{f}_{\boldsymbol{R}_{\boldsymbol{total}}\boldsymbol{,}\boldsymbol{i}_{\boldsymbol{pre}}}$ is the total number of reads at the same site in the recipient. $\boldsymbol{P}_{\boldsymbol{D}_{\boldsymbol{s}},\boldsymbol{i}_{\boldsymbol{pre}}}$ is the sub-dominant variant proportion in the donor at site $\boldsymbol{i}_{\boldsymbol{pre}}$. $\frac{k}{N_{b}}$ is the fraction of the recipient population that carries the sub-dominant variant allele.

${L(N_{b})}_{\boldsymbol{i}_{\boldsymbol{ab}}}$is the likelihood of bottleneck size $N_{b}$ for variants at sites $\boldsymbol{i}_{\boldsymbol{ab}}$ which are absent in the recipient. $\boldsymbol{f}_{\boldsymbol{R}_{\boldsymbol{s}}\boldsymbol{,}\boldsymbol{i}_{\boldsymbol{ab}}}$ is the number of the reads at site$\boldsymbol{i}_{\boldsymbol{ab}}$ with the sub-dominant variant absent in the recipient. $\boldsymbol{f}_{\boldsymbol{R}_{\boldsymbol{total}}\boldsymbol{,}\boldsymbol{i}_{\boldsymbol{ab}}}$ is the total number of base counts of variants that are absent on site $\boldsymbol{i}_{\boldsymbol{ab}}$ in the recipient. $\boldsymbol{P}_{\boldsymbol{D}_{\boldsymbol{s}}\boldsymbol{,}\boldsymbol{i}_{\boldsymbol{ab}}}$ is the sub-dominant proportion in the donor at site $\boldsymbol{i}_{\boldsymbol{ab}}$. $T$is the variant-calling threshold.

2.4 Beta-binomial method:

The only difference between the exact version of the beta-binomial method (Leonard et al., 2017) and the binomial method is that the beta-binomial method models early stochastic dynamics of the viral population in the recipient using a beta distribution. The approximate version of the beta-binomial method does not account for sampling error due to finite NGS coverage; this version of the beta-binomial method assumes that there is no sampling error in NGS data, and the proportions of viral variants can be completely observed in the data set. The two versions of model is:

$$n=n_{pre}+n_{ab}$$

$$log(L(N_{b}))=\sum_{i_{pre}=0}^{n_{pre}} {log(L(N_{b})}_{i_{pre}})+\sum_{i_{ab}=0}^{n_{ab}} {log(L(N_{b})}_{i_{ab}})$$

Approximate version:

Present:

$${L(N_{b})}_{i_{pre}}=\sum_{k=0}^{N_{b}} P\_beta(\boldsymbol{P}_{\boldsymbol{R}_{\boldsymbol{s}}\boldsymbol{,}\boldsymbol{i}_{\boldsymbol{pre}}}|k, N_{b}-k)P\_bin(k|N_{b}, \boldsymbol{P}_{\boldsymbol{D}_{\boldsymbol{s}}\boldsymbol{,}\boldsymbol{i}_{\boldsymbol{pre}}})$$

Absent:

$${L(N_{b})}_{i_{ab}}=\sum_{k=0}^{N_{b}} P\_beta\_cdf(\boldsymbol{P}_{\boldsymbol{R}_{\boldsymbol{s}}\boldsymbol{,}\boldsymbol{i}_{\boldsymbol{ab}}}<T|k, N_{b}-k)P\_bin(k|N_{b}, \boldsymbol{P}_{\boldsymbol{D}_{\boldsymbol{s}}\boldsymbol{,}\boldsymbol{i}_{\boldsymbol{ab}}})$$

Exact version:

Present:

$${L(N_{b})}_{i_{pre}}=\sum_{k=0}^{N_{b}} P\_betabin(\boldsymbol{f}_{\boldsymbol{R}_{\boldsymbol{s}}\boldsymbol{,}\boldsymbol{i}_{\boldsymbol{pre}}}|\boldsymbol{f}_{\boldsymbol{R}_{\boldsymbol{total}}\boldsymbol{,}\boldsymbol{i}_{\boldsymbol{pre}}},k,N_{b}-k)P\_bin(k|N_{b}, \boldsymbol{P}_{\boldsymbol{D}_{\boldsymbol{s}}\boldsymbol{,}\boldsymbol{i}_{\boldsymbol{pre}}})$$

Absent:

$${L(N_{b})}_{i_{ab}}=\sum_{k=0}^{N_{b}} P\_betabin\_cdf(\boldsymbol{f}_{\boldsymbol{R}_{\boldsymbol{s}}\boldsymbol{,}\boldsymbol{i}_{\boldsymbol{ab}}}<T\boldsymbol{f}_{\boldsymbol{R}_{\boldsymbol{total}},\boldsymbol{i}_{\boldsymbol{ab}}}|\boldsymbol{f}_{\boldsymbol{R}_{\boldsymbol{total}},\boldsymbol{i}_{\boldsymbol{ab}}},k, N_{b}-k)P\_bin(k|N_{b}, \boldsymbol{P}_{\boldsymbol{D}_{\boldsymbol{s}}\boldsymbol{,}\boldsymbol{i}_{\boldsymbol{ab}}})$$

The relevant terms in equations have been explained in above sections and are the same as binomial model.

**3.** **Method that requires multiple donor-recipient pairs**

The Wright-Fisher method (Poon et al., 2016; Wright, 1931) differs fundamentally from the other methods in its requirement for multiple transmission pairs. Rather than estimating bottleneck size from the change in variant proportion from donor to recipient, it is sensitive to the variance in this change between transmission pairs, so that smaller bottleneck causes greater inter-pair variation in donor-recipient change. The formula for the Wright-Fisher method for a single site, $i$, is:

$$N_{b,i}=\frac{E\left[ \boldsymbol{P}_{\boldsymbol{D}_{\boldsymbol{d}}\boldsymbol{,i}} \right]E[\boldsymbol{P}_{\boldsymbol{D}_{\boldsymbol{s}}\boldsymbol{,i}}]}{2var(\boldsymbol{P}_{\boldsymbol{D}_{\boldsymbol{s}}\boldsymbol{,i}}-\boldsymbol{P}_{\boldsymbol{R}_{\boldsymbol{s}}\boldsymbol{,i}})}$$

The final bottleneck estimate, $\bar{N_{b}}$, is calculated as the mean across all sites.

$\boldsymbol{P}_{\boldsymbol{D}_{\boldsymbol{d}}\boldsymbol{,i}}$ and $\boldsymbol{P}_{\boldsymbol{D}_{\boldsymbol{s}}\boldsymbol{,i}}$ are the vectors across all transmission pairs of the proportions of the dominant and sub-dominant variants, respectively, in the donor. $\boldsymbol{P}_{\boldsymbol{R}_{\boldsymbol{s}}\boldsymbol{,i}}$ is the sub-dominant variant’s proportions in the recipients across all the transmission pairs.

**Reference**

Emmett, K. J., Lee, A., Khiabanian, H., & Rabadan, R. (2015). High-resolution Genomic Surveillance of 2014 Ebolavirus Using Shared Subclonal Variants. *PLoS Currents*, *7*, ecurrents.outbreaks.c7fd7946ba606c982668a96bcba43c90. <https://doi.org/10.1371/currents.outbreaks.c7fd7946ba606c982668a96bcba43c90>

Kullback, S., & Leibler, R. A. (1951). On information and sufficiency. *The Annals of Mathematical Statistics*, *22*(1), 79–86.

Leonard, A., Weissman, D., Greenbaum, B., Ghedin, E., & Koelle, K. (2017). Transmission Bottleneck Size Estimation from Pathogen Deep-Sequencing Data, with an Application to Human Influenza A Virus. *Journal of Virology*, *91*. <https://doi.org/10.1128/JVI.00171-17>

Poon, L., Song, T., Rosenfeld, R., Lin, X., Rogers, M., Zhou, B., Sebra, R., Halpin, R., Guan, Y., Twaddle, A., DePasse, J., Stockwell, T., Wentworth, D., Holmes, E., Greenbaum, B., Peiris, J. S., Cowling, B., & Ghedin, E. (2016). Quantifying influenza virus diversity and transmission in humans. *Nature Genetics*, *48*. <https://doi.org/10.1038/ng.3479>

Sacristán, S., Díaz, M., Fraile, A., & García-Arenal, F. (2011). Contact transmission of Tobacco mosaic virus: A quantitative analysis of parameters relevant for virus evolution. *Journal of Virology*, *85*(10), 4974–4981. <https://doi.org/10.1128/JVI.00057-11>

Sacristán, S., Malpica, J. M., Fraile, A., & García-Arenal, F. (2003). Estimation of population bottlenecks during systemic movement of tobacco mosaic virus in tobacco plants. *Journal of Virology*, *77*(18), 9906–9911. <https://doi.org/10.1128/jvi.77.18.9906-9911.2003>

Wright, S. (1931). Evolution in mendelian populations. *Genetics*, *16*(2), 97–159.

https://doi.org/10.1093/genetics/16.2.97
